# Recovery of the *N,N*-Dibutylimidazolium Chloride Ionic Liquid from Aqueous Solutions by Electrodialysis Method

**DOI:** 10.3390/ijms23126472

**Published:** 2022-06-09

**Authors:** Dorota Babilas, Anna Kowalik-Klimczak, Anna Mielańczyk

**Affiliations:** 1 Department of Inorganic, Analytical Chemistry and Electrochemistry, Faculty of Chemistry, Silesian University of Technology, B. Krzywoustego 6, 44-100 Gliwice, Poland; 2 Bioeconomy and Eco-Innovation Centre, Łukasiewicz Research Network—The Institute for Sustainable Technologies, Pułaskiego 6/10, 26-600 Radom, Poland; anna.kowalik-klimczak@itee.lukasiewicz.gov.pl; 3 Department of Physical Chemistry and Technology of Polymers, Faculty of Chemistry, Silesian University of Technology, M. Strzody 9, 44-100 Gliwice, Poland; anna.mielanczyk@polsl.pl

**Keywords:** electrodialysis, ionic liquids recovery, 1,3-dialkylimidazolium ionic liquids, *N*,*N*-dibutylimidazolium chloride

## Abstract

Ionic liquids (ILs), named also as liquid salts, are compounds that have unique properties and molecular architecture. ILs are used in various industries; however, due to their toxicity, the ILs’ recovery from the postreaction solutions is also a very important issue. In this paper, the possibility of 1,3-dialkylimidazolium IL, especially the *N*,*N*-dibutylimidazolium chloride ([C_4_C_4_IM]Cl) recovery by using the electrodialysis (ED) method was investigated. The influence of [C_4_C_4_IM]Cl concentration in diluate solution on the ED efficiency was determined. Moreover, the influence of IL on the ion-exchange membranes’ morphology was examined. The recovery of [C_4_C_4_IM]Cl, the [C_4_C_4_IM]Cl flux across membranes, the [C_4_C_4_IM]Cl concentration degree, the energy consumption, and the current efficiency were determined. The results showed that the ED allows for the [C_4_C_4_IM]Cl recovery and concentration from dilute solutions. It was found that the [C_4_C_4_IM]Cl content in the concentrates after ED was above three times higher than in the initial diluate solutions. It was noted that the ED of solutions containing 5–20 g/L [C_4_C_4_IM]Cl allows for ILs recovery in the range of 73.77–92.45% with current efficiency from 68.66% to 92.99%. The [C_4_C_4_IM]Cl recovery depended upon the initial [C_4_C_4_IM]Cl concentration in the working solution. The highest [C_4_C_4_IM]Cl recovery (92.45%) and ED efficiency (92.99%) were obtained when the [C_4_C_4_IM]Cl content in the diluate solution was equal 20 g/L. Presented results proved that ED can be an interesting and effective method for the [C_4_C_4_IM]Cl recovery from the dilute aqueous solutions.

## 1. Introduction

Ionic liquids (ILs) are known as green solvents. ILs are completely composed of ions [1]. Over the past two decades, ILs have gained increasing importance in the industrial sector. Due to their unique properties, they are attractive alternatives to other organic solvents. ILs are characterized by a melting point below 100 °C, high polarity, versatile solubility, high electrical conductivity, high thermal stability, and non-volatility [2]. Due to their properties, ILs can be divided into room temperature ILs, low-temperature ILs, poly-ILs, and magnetic ILs. Room temperature ILs have a liquid or molten state below 100 °C. Low-temperature ILs are used for low-temperature applications and in electrochemical instruments to store energy. In membrane technology, because of their ability and durability to form membranes, poly-ionic liquids can be used. Magnetic ILs are characterized by paramagnetic properties, and easy dispersion in solutions [3,4]. ILs’ applications have a multidisciplinary character. Generally, in chemical processes, ILs are used as reagents, catalysts, and solvents [5]. In biotechnology, ILs can be used for biocatalysis and protein purification [6,7]. In the pharmaceutical industry, the ILs can be applied in drug delivery systems and as active pharmaceutical ingredients [8,9]. Moreover, ILs have been employed in chemical engineering in extraction and separation processes [10]. ILs are often used as additives in synthesis reactions, in the physical processing of polymers, or as reaction media [10].

In last decades, the ILs are of special interest since they have proven their versatility and effectiveness in many areas of chemistry. Their application stems from their unique properties. One of the interesting ILs is *N,N*-dibutylimidazolium chloride ([C_4_C_4_IM]Cl). In the case of [C_4_C_4_IM]Cl, which is an ambient temperature IL, it was successfully used as a “green” solvent and catalyst at once in Friedlander heteroannulation reaction [11], cellulose dissolution, and the dehydration of fructose [12]. The advantages of [C_4_C_4_IM]Cl are good thermal stability, non-volatility, solubility in water, and recyclability.

Unfortunately, due to the good miscibility of ILs with most solvents, their recovery and separation from the organic compound or polymer solutions by traditional separation processes are inefficient. Moreover, the separation of the final product requires numerous unit operations, uses environmentally hazardous reagents, leads to the unfavorable dilution of ILs to very low concentrations during individual stages of processing, and generates an increased amount of harmful waste. Due to the growing generation of wastewater by various industries, the wastewater treatment and recovery of raw materials are very important aspects [1,13,14]. Despite the unique properties of ILs, they can have a negative impact on the environment. In particular, the water-soluble, chemical, and thermal stable ILs occurring in wastewater can contaminate the soil and aquatic environment to a great extent and increase a negative effect on the environment and living organisms [15]. The imidazolium-based ILs indicate toxicity towards the aquatic system, green algae, and microorganisms [10]. In the available literature, it was also found that ILs have the ability to inhibit various enzymes. Therefore, investigations on the removal or recovery of ILs from wastewater and post-reaction mixtures should be developed [16,17,18].

In the available literature, the methods for recovering ILs from wastewater include adsorption, crystallization, distillation, extraction, and membrane processes [19,20]. One of the promising methods of ILs recovery is electrodialysis (ED). ED is an environmentally friendly process for solution desalination, which is easier to scale up than other wastewater treatment techniques such as adsorption or ion-exchange. In addition, ED allows for the recovery and concentration of salts from diluted solutions. In the case of ILs recovery, ED does not require the use of additional solvents. ED is known as a separation process used to separate ions using electrical potential and charged ion-exchange membranes. Thus, because of the electrolyte nature of ILs, ED could be an efficient ILs recovery method [21]. During ED, ions migrate across membranes, anions across the positively charged anion-exchange membranes, and cations across the negatively charged cation-exchange membranes. Thus, the treated solutions are desalted [22,23,24]. ED applications include brackish water desalination, salt pre-concentration, demineralization of food products, and wastewater treatment, especially wastewater from the electroplating industry [25,26,27]. ED can be also applied for ILs recovery from aqueous post-reaction solutions [28]. Effectiveness of electrodialytic ILs recovery depends on the kind of treated ILs—cation or anion. Nowadays, ED is applied for 1-butyl-3-methylimidazolium chloride ([Bmim]Cl), 1-butyl-3-methylimidazolium bromide ([Bmim]Br), 1-butyl-3-methylimidazolium hydrogensulfate ([Bmim]HSO_4_), 1-allyl-3-methylimidazolium chloride ([Amim]Cl), 1-ethyl-3-methylimidazolium chloride ([Emim]Cl), and triethylammonium hydrogen sulfate [TEA][HSO_4_]. It was concluded that the IL recovery highly depends on the kind of ILs, the concentration of ILs in solution, and ED parameters. Depending on the treated ILs solution, alkyl chain length, and ILs concentration, the ILs recovery rate ranges from 40 to 95%. Therefore, it is important to check the effectiveness of ILs recovery for the specific ILs [29,30,31,32,33,34,35].

The purpose of this work is to investigate the possibility and effectiveness of the recovery of *N,N*-dibutylimidazolium chloride ([C_4_C_4_IM]Cl) using the ED method. The influence of [C_4_C_4_IM]Cl concentration in diluate solution on the ED efficiency is discussed in detail. The [C_4_C_4_IM]Cl content in the experimental solutions was selected based on the general ILs content in the wastewater. The recovery and concentration of [C_4_C_4_IM]Cl by the ED method has not yet been demonstrated in the available literature, therefore it can be a novel method for [C_4_C_4_IM]Cl recovery from wastewater.

## 2. Results and Discussion

The aim of this work is to examine the effectiveness of the *N*,*N*-dibutylimidazolium chloride ([C_4_C_4_IM]Cl) recovery using ED method. The influence of the initial concentration of [C_4_C_4_IM]Cl in diluate solution on the ED efficiency was evaluated by the ED effectiveness factors such as recovery ratio, IL concentration rate, IL molar flux across ion-exchange membranes, electric current efficiency, and energy consumption. The IL concentration in feed solution influence on the solution conductivity, electrical resistance, and concentration polarization, as well as simultaneously on the ED efficiency. Thus, four sets of ED experiments with IL concentration in feed solution in the range from 5 to 20 g/L were carried out. The process solutions compositions are presented in Table 1. The experiments were conducted using the method described in Section 3.2.

**Figure 1:**
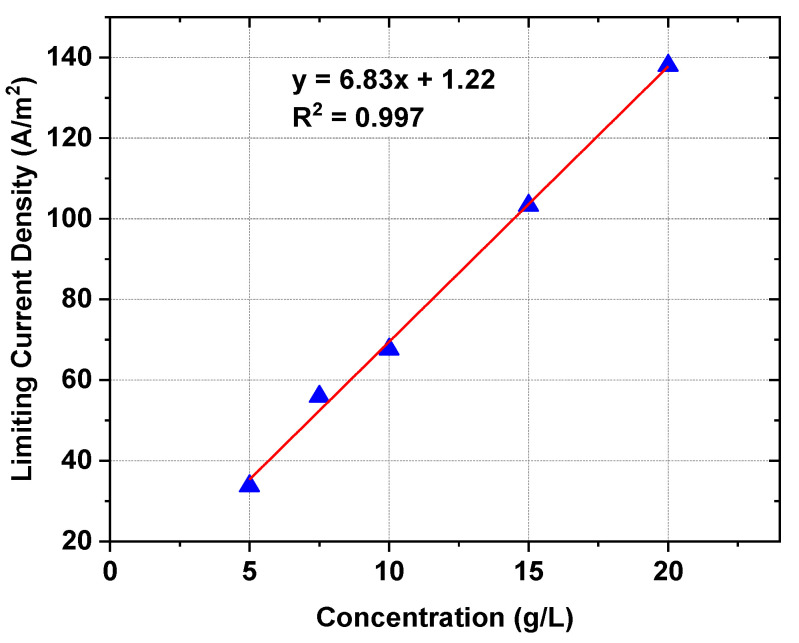
The effect of [C_4_C_4_IM]Cl concentration in the initial diluate on the LCD.

In the first stage of the work, the LCDs were determined. The LCD is a crucial factor in choosing the operational parameters of the ED [36]. LCD also determines the efficiency of the ED. The ED conducted above LCD is characterized by high electrical resistance in the diluate as a result of depletion of the ions in the laminar boundary layer at the ion-exchange membrane surface. The LCD is highly dependent on the concentration of feed solution [37]. The LCD as a function of the [C_4_C_4_IM]Cl concentration in diluate is presented in Figure 1. As was presumed, the LCD increased in a linear manner with increasing [C_4_C_4_IM]Cl content in the feed solution. The determined LCDs were in the range of 34 to 138 A/m^2^. All the ED experiments were conducted below the LCD at constant voltage.

**Figure 2:**
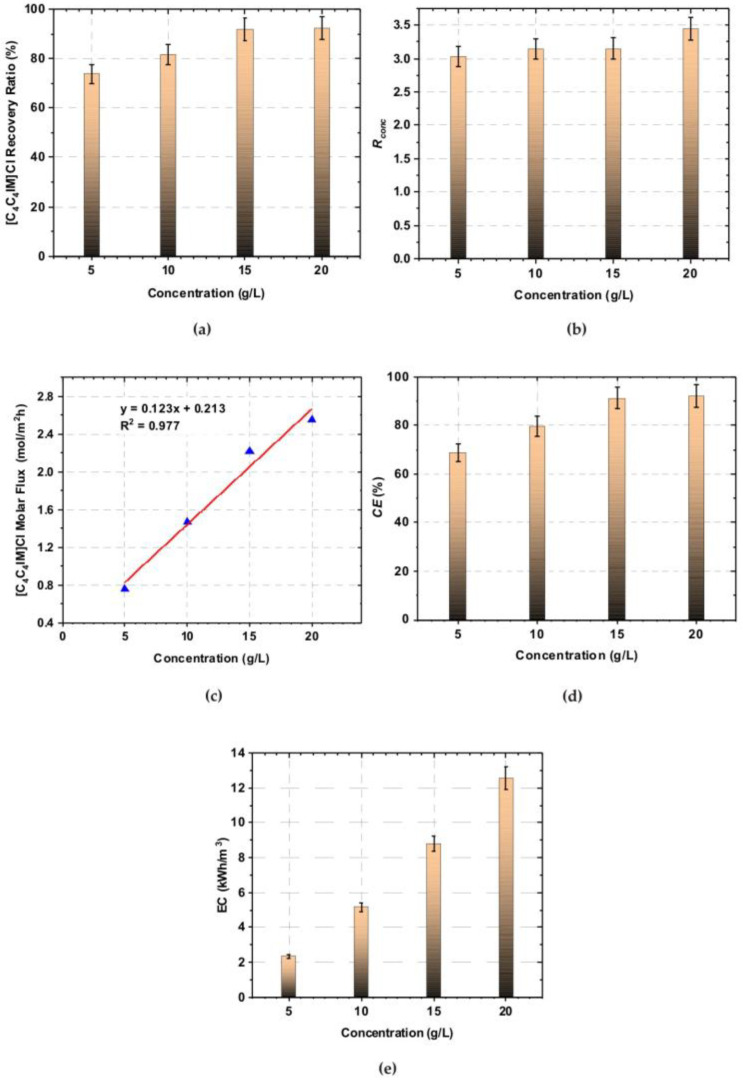
The effect of [C_4_C_4_IM]Cl concentration in the initial diluate on the: (**a**) recovery ratio, (**b**) concentration rate, (**c**) IL molar flux across ion-exchange membranes, (**d**) electrodialysis current efficiency, (**e**) stack energy consumption.

Electrodialytic ILs recovery and concentration degree highly depend on the feed solution concentration. The effect of the initial feed concentration on the [C_4_C_4_IM]Cl recovery effectiveness was presented in Figure 2a–e. Figure 2a shows that the [C_4_C_4_IM]Cl recovery ratio depended on the initial [C_4_C_4_IM]Cl content in the diluate.

It was found that the recovery ratio increased with increasing [C_4_C_4_IM]Cl concentration in the feed solution. The recovery ratio increased from 73.77% to 92.45% for the feed solution of 5 to 20 g/L of [C_4_C_4_IM]Cl, respectively. Thus, it was proved that [C_4_C_4_IM]Cl was effectively removed from diluate solution by the ED method. The recovery ratio reached above 90% for solutions with a [C_4_C_4_IM]Cl content equal to 15 and 20 g/L. The ED at high [C_4_C_4_IM]Cl concentration resulted in a high [C_4_C_4_IM]Cl recovery ratio (in the analyzed concentration range). It is in agreement with research on [BMIM]Cl recovery by ED method [28,29]. In the available literature, it is noted that the initial ILs content in diluate solution has an important effect on the ED effectiveness factors. The results presented in the works [28,29,33] confirmed that the ILs recovery by the ED method increases with increasing ILs concentration in the initial diluate. However, the ILs recovery also is highly dependent on the ILs type and chemical character.

It was also noted that the [C_4_C_4_IM]Cl content in the all concentrates after ED was three times higher than in the feed solution (Figure 2b). Moreover, the obtained results were correlated with [C_4_C_4_IM]Cl molar flux across ion-exchange membranes. It can be clearly seen in Figure 2c that the molar flux of [C_4_C_4_IM]Cl increased with increasing [C_4_C_4_IM]Cl concentration in the feed solution in an almost linear manner. The [C_4_C_4_IM]Cl molar flux across ion-exchange membranes was estimated to be 0.76 mol/m^2^h at the feed solution concentration of 5 g/L [C_4_C_4_IM]Cl and 2.56 mol/m^2^h at the feed solution concentration of 20 g/L [C_4_C_4_IM]Cl.

ED efficiency is also evaluated by electric current efficiency. Current efficiency is defined as the ratio between the current used in the ED stack for effective ion recovery from diluate solution to concentrate and the amount of the total current applied in the ED stack. Current efficiency defines how much of the electric current is effectively used in ion transport across ion-exchange membranes [38,39]. In Figure 2d, the effect of [C_4_C_4_IM]Cl concentration in the initial diluate on the electrodialysis current efficiency is shown. It was found that current efficiency increased with increasing [C_4_C_4_IM]Cl concentration in the initial diluate solution. It can be explained by the reduction of electrical resistance of initial diluate with increasing ILs concentration, and the acceleration of ion transport across membranes. Electric current efficiency increased from 68.66% to a maximum value of 92.99% for the feed solutions in the range from 5 to 20 g/L of [C_4_C_4_IM]Cl.

The current efficiency increased linearly for diluates with IL concentrations ranging from 5 to 15 g/L. However, above a concentration of 15 g/L, the current efficiency increases slightly. It was concluded that obtained current efficiencies in the examined range are very satisfactory in comparison to values presented in other works about ILs recovery by ED methods. Current efficiency of the electrodialytic recovery of [BMIM]Cl increased from 37.7% to 70.7% for initial [BMIM]Cl content in feed solutions from 2.24 to 6.90 g/L [30]. In another work [29], current efficiency of [BMIM]Cl recovery was over 70% under the condition of IL concentration equaled 34.94 g/L (0.2 mol/L).

As is shown in Figure 2e, the initial [C_4_C_4_IM]Cl content in diluate also has an effect on the energy consumption. It was observed that the stack energy consumption highly depended on the initial [C_4_C_4_IM]Cl content in the diluate. It can be clearly seen that energy consumption increased linearly with increasing of [C_4_C_4_IM]Cl concentration in the initial diluate solution. When the initial concentration of [C_4_C_4_IM]Cl increased from 5 to 20 g/L, the energy consumption increased from 2.35 to 12.57 kWh/m^3^, respectively.

The obtained results confirmed, that the electrodialytic [C_4_C_4_IM]Cl recovery is influenced by feed solution concentration. The best ED performance was obtained when the [C_4_C_4_IM]Cl content in the initial diluate was 15 and 20 g/L. When the concentration of [C_4_C_4_IM]Cl in the initial diluate was 15 g/L, the [C_4_C_4_IM]Cl recovery ratio, the [C_4_C_4_IM]Cl concentration rate, the electric current efficiency, as well as energy consumption were 91.87%, 3.15, 91.40%, and 8.77 kWh/m^3^, respectively. It was also noted that when in the initial diluate the [C_4_C_4_IM]Cl concentration was 20 g/L, the 3.45-fold concentration degree can be achieved with 92.45% [C_4_C_4_IM]Cl recovery ratio and current efficiency of 92.99%.

**Figure 3:**
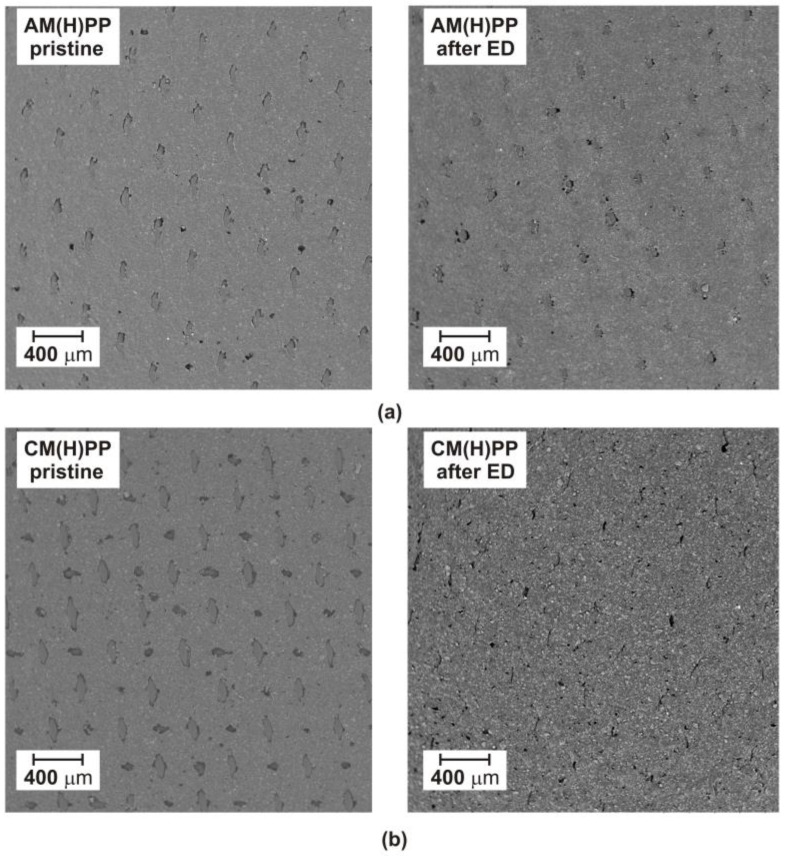
SEM micrograph of the tested heterogeneous ion-exchange membranes before and after ED: (**a**) AM(H)PP; (**b**) CM(H)PP membrane.

One of the disadvantages of the electrodialytic IL recovery is membrane fouling. Membrane fouling can be described as agglomerations of molecules, inorganic, and organic compounds in the membrane pores or on the membrane surface. Membrane fouling causes reduced membrane separation efficiency. Membrane fouling can be limited by the linear flow velocity of feed solution, ED operational parameters, and the ED module chemical cleaning after process [39,40]. In Figure 3, the SEM micrographs of the tested heterogeneous ion-exchange membranes before and after ED are presented. The AM(H)PP and CM(H)PP membranes were fabricated by a pressing method from polypropylene (as an inert polymer membrane matrix) and ion-exchange resin particles. In Figure 3, the morphology of the pristine AM(H)PP and CM(H)PP membranes are presented. The morphology of pristine membranes are inhomogeneous. The pores, ion-exchange resin grains, and reinforcing net are clearly observed on the pristine membranes’ surface (Figure 3). It was also found that the morphology of the anion-exchange membranes’ AM(H)PP did not differ from that of the pristine AM(H)PP. On the both AM(H)PP membrane surface micrographs, the ion-exchange grains in the polymer membrane matrix, the reinforcing net, and the pores can be clearly seen. However, in the case of the cation-exchange membranes’ (CM(H)PP) SEM micrographs, it can be noted that the fouling occurred. It was found that the morphology of the CM(H)PP differs from that of the pristine CM(H)PP. The layer on the CM(H)PP membrane surface is caused probably by [C_4_C_4_IM]^+^. The ionic radius of [C_4_C_4_IM]^+^ and the alkyl chain length also influence the fouling ability. As the ion radius increases, the probability of fouling increases. The size of [C_4_C_4_IM]^+^ is larger than that of [C_4_MIM]^+^, and in consequence higher fouling of the CM(H)PP membrane was observed in comparison to the ED of [BMIM]Cl [41]. The pores on the surface of the CM(H)PP membrane after ED are clearly smaller (Figure 3). The reinforcing net is also less visible. Fouling of the CM(H)PP membranes can reduce the flux and increase energy consumption [39]. Although some kind of the cation-exchange membranes fouling was observed, the obtained results confirmed that the ED performance was not affected, and the ED can be applied as an efficient [C_4_C_4_IM]Cl recovery method from wastewater and aqueous solutions. A very important aspect is the cleaning of the membranes after ED. Membranes can be cleaned with distilled water [39], 0.35 wt% HCl [30], as well as 0.4 wt% NaOH [30].

## 3. Materials and Methods

### 3.1. Experimental Solutions

Experiments were conducted using the model diluate and concentrate solutions containing [C_4_C_4_IM]Cl. The concentration of [C_4_C_4_IM]Cl in the diluate and concentrate solutions was in the range of 5–20 g/L. The [C_4_C_4_IM]Cl content in solutions was selected based on the general ILs content in the wastewater. Frequently, the content of IL in the wastewater and post-reaction solution is in the range of 2.24 to 35 g/L [29,30,33].

The [C_4_C_4_IM]Cl was synthesized as follows: 5 mL of butylimidazole (114 mmol), 14.2 mL of chlorobutane (136.8 mmol), and 30 mL of toluene were put in a round bottom flask, which was then placed in an oil bath at 120 °C and equipped with a reflux condenser. The reaction was carried out for 24 h. After a designated time, the mixture was cooled to room temperature. The upper layer was collected with a syringe. Toluene from the bottom layer was evaporated in a rotary evaporator with a water bath temperature of 100 °C. The ionic liquid was dissolved in methylene chloride, and activated carbon was added to remove possible impurities. The whole solution was passed through a filter and again evaporated in a rotary evaporator with a water bath temperature of 50 °C. Finally, the ionic liquid was dried to get rid of water and not distilled toluene. ^1^H NMR (400 MHz, CDCl3) δ [ppm]: 0.95–1.00 (t, 6H, >NCH_2_CH_2_CH_2_CH_3_), 1.35–1.43 (sextet, 4H, >NCH_2_CH_2_CH_2_CH_3_), 1.88–1.95 (quintet, 4H, >NCH_2_CH_2_CH_2_CH_3_), 4.35–4.40 (t, 4H, >NCH_2_CH_2_CH_2_CH_3_), 7.43 (d, 2H, position 4 and 5 in the ring N-CH = CH-N) 10.92 (s, 1H, position 2 in the ring N-CH = N).

The 0.1 M H_2_SO_4_ (Avantor Performance Materials, Gliwice, Poland) solution was used as the electrode rinse solution. All experimental solutions were prepared using deionized water (Millipore Elix 10 system, Darmstadt, Germany).

### 3.2. Experimental Set-Up

**Figure 4:**
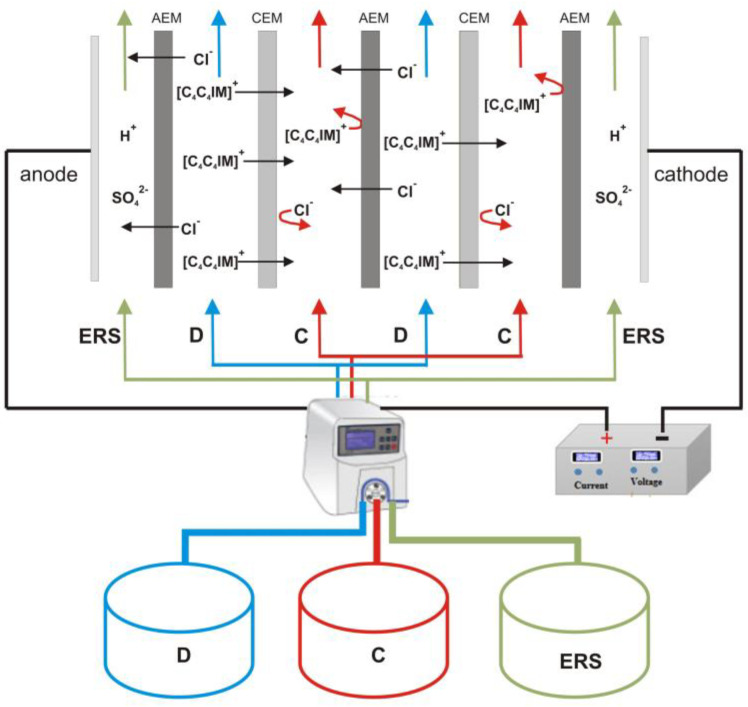
Experimental ED set-up for [C_4_C_4_IM]Cl recovery. D—diluate, C—concentrate, ERS—electrode rinse solution, AEM—anion-exchange membrane, CEM—cation-exchange membrane.

The electrodialytic [C_4_C_4_IM]Cl recovery was carried out at room temperature using the experimental set-up consisting of the EDR-Z/10-0.8 module (MemBrain, Straz pod Ralskem, Czech Republic) with two pairs of the heterogeneous ion-exchange membranes AM(H)PP–CM(H)PP (Mega a.s., Straz pod Ralskem, Czech Republic) in the ED stack. An effective area of the single membrane was 64 cm^2^. The ED module was connected to a programmable power supply (KORAD KA3010, KORAD Technology Co., Ltd., Dongguan, China). The experiments were performed under constant voltage conditions, which was a maximum value determined by the limiting current density test. The electric current of the electrodialysis system was recorded every 1 min. The electrodialyzer was connected with three tanks named diluate, concentrate, and electrode rinse solution. All process solutions were recirculated using a peristaltic pump (MCP Standard Ismatec, Cole-Parmer, Wertheim, Germany) at a rate corresponding to the linear flow velocity of 2 cm/s. The initial volume of diluate was 300 mL, the initial volume of concentrate was 100 mL, and the volume of the electrode rinse solution was 250 mL. Thus, the diluate-to-concentrate volume ratio was equal to 3. The experiments were conducted until the diluate conductivity dropped to 10% value of initial diluate conductivity, which was monitored using two CX-461 conductivity meters (Elmetron, Zabrze, Poland). Experimental ED set-up for [C_4_C_4_IM]Cl recovery is presented in Figure 4. All experiments were replicated three times.

### 3.3. Membranes

AM(H)PP–CM(H)PP ion-exchange membranes were used in the experiments. The CM(H)PP and AM(H)PP membranes were manufactured by Mega a.s. (Straz pod Ralskem, Czech Republic). The tested membranes comprised an ion exchange resin incorporated within a binder. Before and after the ED experiments, the surface morphology of the tested membranes was investigated using a scanning electron microscope (Hitachi TM3000 table-top TM series, Tokyo, Japan), equipped with a backscattered electron (BSE) detector.

### 3.4. Limiting Current Density (LCD)

The LCDs for solutions with [C_4_C_4_IM]Cl concentrations of 5, 7.5, 10, 15, and 20 g/L were determined by the Cowan–Brown method [36]. During the LCDs determination, the applied voltage was increased stepwisely at a speed of 0.5 V/min until the ED cell potential drop reached 20 V. The LCDs were determined from the relationship between the current and the corresponding potential. Therefore, the ED stack resistance–reciprocal current curves for the LCD assessment were drawn.

### 3.5. Analytical Methods

The concentrations of [C_4_C_4_IM]Cl in diluate and concentrate solutions were analyzed using a UV-VIS spectrophotometer (Varian Cary 50 Scan, Agilent, Santa Clara, CA, USA). The maximum absorption wavelength for the [C_4_C_4_IM]^+^ cation was 211.50 nm. The concentration of [C_4_C_4_IM]Cl was determined based on the standard curve between the concentration and absorbance of [C_4_C_4_IM]Cl. The standard curve between the concentration and absorbance of [C_4_C_4_IM]Cl is presented in Figure S1.

### 3.6. ED Experiments Data Analysis

To estimate the [C_4_C_4_IM]Cl recovery effectiveness, some crucial factors such as the [C_4_C_4_IM]Cl recovery ratio ($R_{[C_{4}C_{4}IM]Cl}$), the [C_4_C_4_IM]Cl concentration rate ($R_{conc}$), the [C_4_C_4_IM]Cl molar flux across ion-exchange membranes ($J_{[C_{4}C_{4}IM]Cl}$), the electric current efficiency ($CE_{[C_{4}C_{4}IM]Cl}$), as well as energy consumption (*EC*) were calculated using the following Equations (1)–(5), respectively:

(1) $$R_{[C_{4}C_{4}IM]Cl}=\frac{m_{IL,t}^{conc}}{m_{IL,0}^{dil}}\cdot 100\%$$

where:

▪ $m_{IL,0}^{dil}$—the initial mass of the [C_4_C_4_IM]Cl in the diluate before ED, [g],
▪ $m_{IL,t}^{conc}$—the increase in the [C_4_C_4_IM]Cl mass in the concentrate after ED, [g].

(2) $$R_{conc}=\frac{C_{IL,t}^{conc_{}}}{C_{IL,0}^{dil_{}}}\cdot 100\%\;$$

where:

▪ $C_{IL,0}^{dil}$—the initial concentration of the [C_4_C_4_IM]Cl in the diluate solution before ED, [g/L],
▪ $C_{IL,t}^{conc_{}}$—the final concentration of the [C_4_C_4_IM]Cl in the concentrate solution after ED, [g/L].

(3) $$J_{[C_{4}C_{4}IM]Cl}=\frac{\left(V_{t}^{conc}\cdot C_{IL,t}^{conc}\right)-\left(V_{0}^{conc}\cdot C_{IL,0}^{conc}\right)}{M_{[C_{4}C_{4}IM]Cl}\cdot A\cdot t}\;$$

where:

▪ $V_{t}^{conc}$—the volume of the concentrate solution after ED, [L],
▪ $V_{0}^{conc}$—the volume of the concentrate solution before ED, [L],
▪ $C_{IL,t}^{conc}$—the concentration of the [C_4_C_4_IM]Cl in the concentrate solution after ED, [g/L],
▪ $C_{IL,0}^{conc}$—the concentration of the [C_4_C_4_IM]Cl in the concentrate solution before ED, [g/L],
▪ $M_{[C_{4}C_{4}IM]Cl}$—the molar mass of [C_4_C_4_IM]Cl, [g/mol],
▪ A—the active membrane surface area, [m^2^],
▪ t—ED time, [h].

(4) $$CE_{[C_{4}C_{4}IM]Cl}=\frac{F\cdot z\cdot \frac{C_{IL,t}^{conc_{}}}{M_{[C_{4}C_{4}IM]Cl}}\cdot V_{t}^{conc}}{n\cdot \int_{0}^{t}I(t)dt}\cdot 100\%$$

where:

▪ *F*—the Faraday constant (96,485 C/mol),
▪ *z*—the charge number of [C_4_C_4_IM]^+^,
▪ $V_{t}^{conc}$—the volume of the concentrate solution after ED, [L],
▪ $C_{IL,t}^{conc_{}}$—the concentration of the [C_4_C_4_IM]Cl in the concentrate solution after ED, [g/L],
▪ $M_{[C_{4}C_{4}IM]Cl}$—the molar mass of [C_4_C_4_IM]Cl, [g/mol],
▪ *n*—the number of membrane pairs,
▪ *I*—the electric current, [A].

(5) $$EC=\frac{U\cdot \int_{0}^{t}I(t)dt}{V_{0}^{dil}}$$

where:

▪ *EC*—the energy consumption, [kWh/m^3^],
▪ *U*—the applied voltage, [V],
▪ *I*—the electric current, [A],
▪ $V_{0}^{dil}$—the initial diluate volume, [L].

## 4. Conclusions

In this work, the possibility and effectiveness of *N,N*-dibutylimidazolium chloride recovery using the ED method were discussed. It was concluded that ED can be applied as an efficient [C_4_C_4_IM]Cl recovery method from wastewater and aqueous solutions. It was proved that the [C_4_C_4_IM]Cl content in the feed solution influences the ED performances. The recovery ratio, the [C_4_C_4_IM]Cl molar flux, and the electric current efficiency increase with increasing the concentration of [C_4_C_4_IM]Cl in the feed solution. Moreover, the energy consumption also highly depends on the initial [C_4_C_4_IM]Cl content in diluate. Energy consumption increases linearly with increasing of the [C_4_C_4_IM]Cl concentration in the initial diluate solution.

It was also found that in the examined [C_4_C_4_IM]Cl concentration range, the best ED performance can be obtained when the [C_4_C_4_IM]Cl content in the initial diluate is 15 and 20 g/L. When in the initial diluate the [C_4_C_4_IM]Cl concentration is 20 g/L, the 3.45-fold concentration degree can be achieved with 92.45% [C_4_C_4_IM]Cl recovery ratio and current efficiency 92.99%.

The ILs concentration in feed solution influences the solution conductivity, electrical resistance, and concentration polarization, as well as simultaneously the ED efficiency. It was found that ED efficiency increased with increasing [C_4_C_4_IM]Cl concentration in the initial diluate solution (in the examined range). It was explained by the reduction of electrical resistance of the initial diluate with increasing ILs concentration, and the acceleration of ions transport across membranes.

## Figures and Tables

**Table 1:** The experimental solutions composition.

Exp. No.	Initial Diluate	Initial Concentrate	Electrode Rinse Solution
1.	300 mL of 5 g/L [C_4_C_4_IM]Cl	100 mL of 5 g/L [C_4_C_4_IM]Cl	250 mL of 0.1 M H_2_SO_4_
2.	300 mL of 10 g/L [C_4_C_4_IM]Cl	100 mL of 10 g/L [C_4_C_4_IM]Cl	250 mL of 0.1 M H_2_SO_4_
3.	300 mL of 15 g/L [C_4_C_4_IM]Cl	100 mL of 15 g/L [C_4_C_4_IM]Cl	250 mL of 0.1 M H_2_SO_4_
4.	300 mL of 20 g/L [C_4_C_4_IM]Cl	100 mL of 20 g/L [C_4_C_4_IM]Cl	250 mL of 0.1 M H_2_SO_4_

## Data Availability

The data presented in this study are available on request from the corresponding author.

## Supplementary Materials

The following supporting information can be downloaded at: https://www.mdpi.com/article/10.3390/ijms23126472/s1.
