# Supplementary material for: Recovery of the N,N-Dibutylimidazolium Chloride Ionic Liquid from Aqueous Solutions by Electrodialysis Method
Source: Int J Mol Sci. 2022 Jun 9;23(12):6472. doi: 10.3390/ijms23126472 (PMC9224464; doi:10.3390/ijms23126472)
Supplement: Supplementary file 1 [file ijms-23-06472-s001.zip › ijms-1746607-supplementary.pdf]

Supplementary materials

Recovery of the *N,N*-Dibutylimidazolium Chloride Ionic Liquid from Aqueous Solutions by Electrodialysis Method

Dorota Babilas [1,*], Anna Kowalik-Klimczak [2] and Anna Mielańczyk [3]

[1] Department of Inorganic, Analytical Chemistry and Electrochemistry, Faculty of Chemistry, Silesian University of Technology, B. Krzywoustego 6, 44-100 Gliwice, Poland
[2] Bioeconomy and Eco-Innovation Centre, Łukasiewicz Research Network—The Institute for Sustainable Technologies, Pułaskiego 6/10, 26-600 Radom, Poland
[3] Department of Physical Chemistry and Technology of Polymers, Faculty of Chemistry, Silesian University of Technology, M. Strzody 9, 44-100 Gliwice, Poland
* Correspondence: dorota.babilas@polsl.pl; Tel.: +48-32-237-24-90

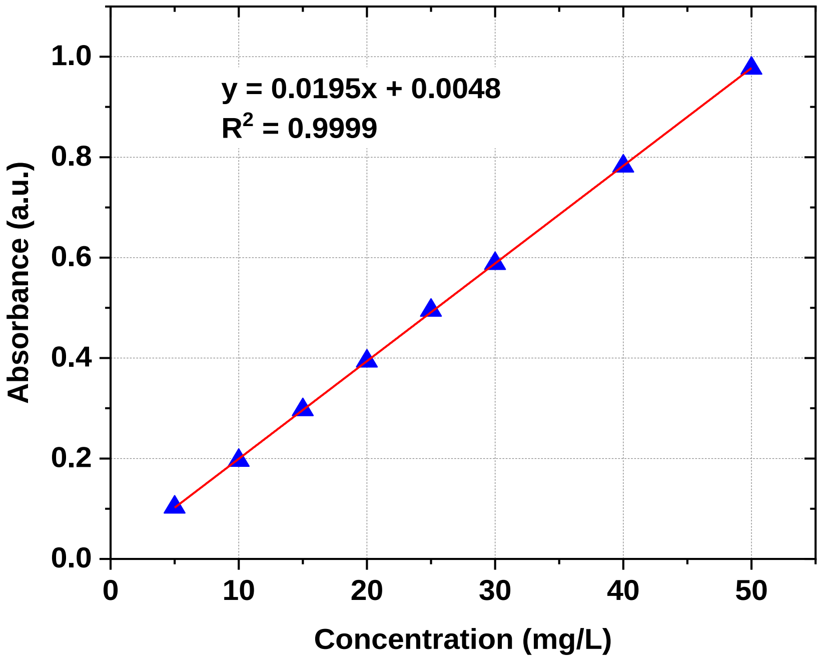

Figure S1. The standard curve between the concentration and absorbance of [C_4C_4IM]Cl.
